# LncRNA NNT-AS1 promotes lung squamous cell carcinoma progression by regulating the miR-22/FOXM1 axis

**DOI:** 10.1186/s11658-020-00227-8

**Published:** 2020-05-29

**Authors:** Jing Ma, Guanbin Qi, Lei Li

**Affiliations:** Department of Respiratory and Critical Care Medicine, Huaihe Hospital of Henan University, NO.115 Ximen Street, Kaifeng City, Henan Province, Kaifeng, 475000 Henan China

**Keywords:** NNT-AS1, miR-22, FOXM1, LUSC, Progression

## Abstract

**Background:**

Recent studies have revealed that dysregulated expression of long non-coding RNA nicotinamide nucleotide transhydrogenase antisense RNA 1 (lncRNA NNT-AS1) is associated with cell tumorigenicity in non-small cell lung cancer. However, the exact molecular mechanisms of NNT-AS1 in lung squamous cell carcinoma (LUSC) remain largely unknown.

**Methods:**

The expression of NNT-AS1, microRNA (miR)-22 and Forkhead box protein M1 (FOXM1) was measured using quantitative real-time polymerase chain reaction or western blot, respectively. The interaction between miR-22 and NNT-AS1 or FOXM1 was confirmed using a dual-luciferase reporter assay and RNA immunoprecipitation assay. Cell migration and invasion abilities were measured by Transwell assay. Flow cytometry was used to detect apoptotic cells.

**Results:**

NNT-AS1 and FOXM1 were up-regulated but miR-22 was down-regulated in LUSC tissues and cell lines. NNT-AS1 was a sponge of miR-22, and NNT-AS1 deletion suppressed the migration and invasion but induced apoptosis in LUSC cells. FOXM1 was a target of miR-22, and overexpression of miR-22 inhibited cell carcinogenesis in LUSC by targeting FOXM1. Additionally, NNT-AS1 could directly regulate FOXM1 expression by binding to miR-22 in LUSC cells.

**Conclusion:**

LncRNA NNT-AS1 contributes to cell carcinogenesis in LUSC by regulating the miR-22/FOXM1 axis, providing a novel insight into the pathogenesis of LUSC and a new potential therapeutic target for LUSC treatment.

## Background

Lung cancer remains the leading cause of cancer-associated mortality worldwide. Non-small cell lung cancer (NSCLC) is one of the main sub-types of lung cancer, accounting for around 80–85% of all lung cancers [1, 2]. NSCLC is histologically categorized into lung squamous cell carcinoma (LUSC), lung adenocarcinoma, large cell carcinoma and neuroendocrine cancer [3]. Among these, LUSC is a common type of NSCLC with a high risk of metastasis and relapse [4]. Thus, further investigations to identify therapeutic biomarkers and molecular mechanisms on the pathogenesis of LUSC are necessary.

Long non-coding RNAs (lncRNAs) is a kind of non-coding RNA of more than 200 nucleotides in length without protein-coding capacity [5]. Increasing evidence has identified that lncRNAs are novel master contributors in regulating malignant physiological or pathological cellular processes, such as carcinogenesis, metastasis, angiogenesis, metabolism, and so on [6, 7]. Also, lncRNAs have been identified to play important functions in the initiation and development of various malignancies, including lung cancer [8, 9]. LncRNA nicotinamide nucleotide transhydrogenase antisense RNA 1 (lncRNA NNT-AS1) is a newly detected cancer-related lncRNA. Recently, dysregulated expression of NNT-AS1 has been identified in several solid tumors, such as gastric, cervical, breast and ovarian cancer, and is associated with cancer tumorigenesis, metastasis, and progression of tumors [10–13]. Nevertheless, the role of NNT-AS1 in LUSC remains largely unknown.

MicroRNAs (miRNAs) are defined as small non-coding RNA molecules, which control gene expression via interacting with the mRNAs of target genes [14, 15]. To date, many miRNAs are known to be involved in the development of cancer through acting as anti-tumor or carcinogenic agents in LUSC, and miRNAs are potential biomarkers for diagnosis and prognosis and therapeutic targets for LUSC [16–18]. LncRNAs contain multiple miRNA-binding sites that have been investigated as competing endogenous RNAs (ceRNA) to indirectly modulate mRNAs through binding to miRNAs [19, 20]. The important roles of the lncRNA-miRNA-mRNA regulatory network and a protein-protein interaction network in lung cancer development have also been identified [21, 22]. Therefore, it is necessary to identify the important regulatory pathway or therapeutic biomarkers according to lncRNA-miRNA-mRNA networks.

In the present study, we aimed to investigate the biological effects of NNT-AS1 in LUSC cell carcinogenesis, and to explore the interaction network of NNT-AS1 in LUSC development. This study may provide novel therapeutic targets for LUSC treatment.

## Materials and methods

### Patients and specimens

This study was permitted by the Ethics Committee of Huaihe Hospital of Henan University and informed consent was obtained from all patients. Tumor specimens and paratumor samples were obtained from 46 paired LUSC patients who underwent surgical resections at Huaihe Hospital of Henan University and were immediately stored at − 80 °C until further analysis. None of the patients received any preoperative treatment and all underwent histopathological examination. Moreover, all procedures carried out in studies involving human participants were in accordance with the ethical standards of the Ethics Committee of Huaihe Hospital of Henan University, and with the 1964 Helsinki Declaration and its later amendments or comparable ethical standards.

### Cell culture

Human bronchial epithelial cell line 16HBE and LUSC cell lines (H1703, SW900, H2170 and U1752) were obtained from Shanghai Academy of Life Science (Shanghai, China), and were cultured in Dulbecco’s modified Eagle’s medium (DMEM; Gibco, Carlsbad, CA, USA) harboring 10% fetal bovine serum and 1% penicillin-streptomycin solution (Gibco) at 37 °C with 5% CO_2_.

### Cell transfection

The small interfering RNA (siRNA) sequences targeting NNT-AS1 (si-NNT-AS1), siRNA negative control (si-con), pcDNA3.1-NNT-AS1 overexpression vector (NNT-AS1), pcDNA3.1-Forkhead box protein M1 (FOXM1) overexpression vector (FOXM1), and pcDNA3.1 empty vector (vector) were synthesized by Invitrogen (Carlsbad, CA, USA). The miR-22 mimic (miR-22), miR-22 inhibitor (in-miR-22) and their corresponding negative control (miR-con or in-miR-con) were obtained from RIBOBIO (Guangzhou, China). All oligonucleotides or vectors were respectively transfected into H1703 and H2170 cells using Lipofectamine 3000 (Invitrogen) according to the standard procedure.

### Quantitative real-time polymerase chain reaction (qRT-PCR)

Total RNA was extracted using a TRIzol kit (Invitrogen) according to the standard protocol. For NNT-AS1 and FOXM1 detection, total RNA was reversely transcribed into complementary DNA (cDNA) using a high-capacity cDNA Reverse Transcription kit (Applied Biosystems, Foster City, CA, USA), and then qPCR was carried out with SYBR Premix Ex Taq (Qiagen, Valencia, CA, USA). As to miR-22 detection, cDNA was synthesized using a TaqMan Reverse Transcription Reagents Kit (Applied Biosystems) and amplified with TransScript Green One-Step qRT-PCR SuperMix (Qiagen). The relative expression was analyzed by the 2^-△△Ct^ method and normalized byglyceraldehyde-3-phosphate dehydrogenase (GADPH) or U6 small nuclear RNA (snRNA). The specific primer sequences used were as follows: NNT-AS1, F: 5′-TCTCCTAAGTCGAGGACTAGC-3′, R: 5′-AGGCACTCACTAGCATCACGCT-3′;FOXM1, F: 5′-GGAGCAGCGACAGGTTAAGG-3′, R: 5′-GTTGATGGCGAATTG TATCATGG-3′; miR-22, F: 5′-GGGGGATCCCTGGGGCAGGACCCT-3′, R: 5′-GGGGAATTCAACGTATCATCCACCC-3′; U6: F:5′-GCTTCGGCAGCACATATACTAAAAT-3′, R: 5′-CGCTTCACGAATTTGCGTGTCAT-3′; GAPDH, F: 5′-AACTTTGGCATTGTGGAAGG-3′, R: 5′-ACACATTGGGGGTAGGAACA-3′.

### Dual-luciferase reporter assay

The WT or MUT NNT-AS1/FOXM1 3′UTR containing a miR-22 binding site was amplified and cloned into the pmirGLO Vector (Promega, Shanghai, China). Then H1703 and H2170 cells were seeded in 24-well plates and co-transfected with miR-22 or miR-con and the corresponding luciferase reporter vectors using Lipofectamine 2000 for 48 h. Subsequently, a dual-luciferase reporter assay kit (Promega) was used to measure the changes in relative luciferase activity.

### RNA immunoprecipitation (RIP) assay

Transfected cells were lysed, and the lysate was incubated with magnetic beads coated with anti-Ago2 (Millipore, Billerica, MA, USA) or IgG antibody (Abcam, Cambridge, MA, USA). Finally, the enrichment of NNT-AS1 or FOXM1 was examined using qRT-PCR.

### Cell migration and invasion

For detection of migrated cells, top chambers were loaded with cell suspension (5 × 10^4^ cells). Then 500 μL of DMEM mixed with 10% FBS was added to the bottom chambers. After incubation for 48 h at 37 °C, migrated cells were fixed with methanol and stained with 0.1% crystal violet. For detection of invaded cells, the upper chamber membranes were pre-coated with Matrigel (Becton Dickinson, Franklin Lakes, NJ, USA) and the measurement method was similar to the steps of cell migration. Finally, cells in five randomly selected fields were counted with a microscope.

### Cell apoptosis assay

Cell apoptosis was analyzed using an Annexin V-FITC/PI apoptosis detection kit (BD Biosciences, San Jose, CA, USA) according to the supplier’s direction. Transfected cells were collected and re-suspended with binding buffer, followed by staining with 10 μL of fluorescein isothiocyanate (FITC) annexin V and propidium iodide (PI). Finally, apoptotic cells were measured by flow cytometry.

### Western blot

Proteins were isolated using RIPA lysis buffer (Beyotime, Shanghai, China) and then were quantified by a bicinchoninic acid (BCA) method following the standard protocol. An equal amount of the extracts was treated with 10% sodium dodecyl sulfate-polyacrylamide gel electrophoresis (SDS-PAGE) and transferred onto polyvinylidene difluoride membranes (Millipore). Subsequently, the membranes were incubated with specific primary antibodies against FOXM1 (1:1000, 20,459, Cell Signaling Technology, Boston, MA, USA) and β-actin (1:1000, 4967, Cell Signaling Technology), followed by incubation with HRP-conjugated secondary antibody (1:1000; ab9482; Abcam). Finally, protein signals were visualized using an ECL method.

### Statistical analysis

Experimental data from three independent experiments were expressed as the mean ± standard deviation (SD) and analyzed using GraphPad Prism 7 software (GraphPad Inc., San Diego, CA, USA). The differences between two groups were analyzed by Student’s *t* test and multiple comparisons were performed by one-way analysis of variance (ANOVA). The correlation analysis was performed using Spearman’s correlation test. *P* < 0.05 was regarded as statistically significant.

## Results

### NNT-AS1 and FOXM1 are up-regulated but miR-22 is down-regulated in LUSC tissues and cell lines

**Fig. 1:**
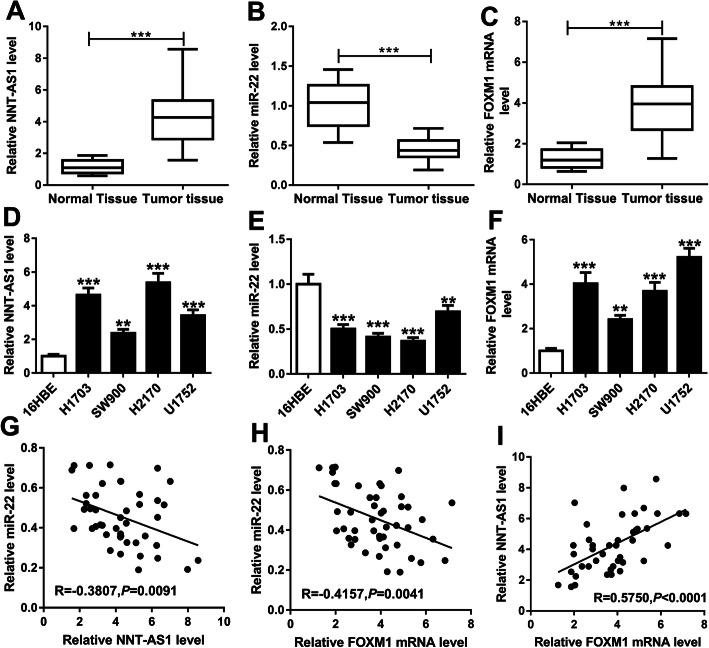
NNT-AS1 and FOXM1 are up-regulated but miR-22 is down-regulated in LUSC tissues and cell lines. **a**-**c** Levels of NNT-AS1, miR-22 and FOXM1 were detected in LUSC tissues and non-tumor tissues using qRT-PCR. **d**-**f** Levels of NNT-AS1, miR-22 and FOXM1 in human bronchial epithelial cell line 16HBE and LUSC cell lines (H1703, SW900, H2170 and U1752) were detected using qRT-PCR. **g**-**i** The expression association among NNT-AS1, miR-22 and FOXM1 was analyzed using Spearman’s correlation test. *P < 0.05, **P < 0.01, ***P < 0.001.

To explore the potential biological functions of NNT-AS1, miR-22 and FOXM1 involved in LUSC carcinogenesis, we detected the levels of NNT-AS1, miR-22 and FOXM1 in 46 paired LUSC tissues and paratumor tissues. Subsequently, qRT-PCR results showed that, compared with the non-tumor tissues, NNT-AS1 and FOXM1 were up-regulated, while miR-22 was down-regulated in LUSC tissues (Fig. 1a-c). The same expression changes were also found in the LUSC cell lines compared with the human bronchial epithelial cell line 16HBE (Fig. 1d-f). After that, expression association was analyzed and a negative correlation between miR-22 and NNT-AS1 (*R* = -0.3807, *P* = 0.0091) or FOXM1 (*R* = -0.4157, *P* = 0.0041), and a positive correlation between NNT-AS1 and FOXM1 were observed (*R* = 0.5750, *P* < 0.0001) (Fig. 1g-i). Therefore, we suspected that NNT-AS1, miR-22 and FOXM1 might be associated with the progression of LUSC.

### NNT-AS1 is a sponge of miR-22

**Fig. 2:**
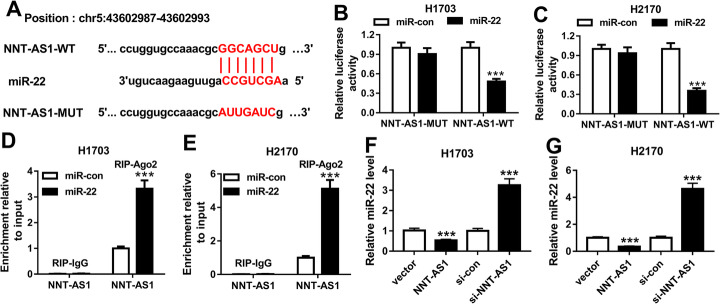
NNT-AS1 is a sponge of miR-22. **a** The binding site between miR-22 and NNT-AS1 is shown. **b**, **c** Luciferase activity was examined in H1703 and H2170 cells co-transfected with NNT-AS1-WT, or NNT-AS1-MUT and miR-22, or miR-con. **d**, **e** Enrichment of NNT-AS1 was analyzed in H1703 and H2170 cells transfected with miR-con or miR-22 after RIP. **f**, **g** The level of miR-22 was detected in H1703 and H2170 cells transfected with vector, NNT-AS1, si-con, or si-NNT-AS1 using qRT-PCR. *P < 0.05, **P < 0.01, ***P < 0.001

To detect the relationship between NNT-AS1 and miR-22, StarBase software was used. As shown in Fig. 2a, miR-22 was identified to have a binding site with NNT-AS1. Moreover, the transfection efficiency of miR-22 overexpression, NNT-AS1 overexpression, and NNT-AS1 deletion is shown and examined in Sup. Fig. 1. To verify this prediction, the dual-luciferase reporter assay was performed. The results indicated that miR-22 overexpression reduced the luciferase activities of the NNT-AS1-WT reporter vector compared to the control group, while there was no obvious change in NNT-AS1-MUT reporter after miR-22 transfection in H1703 and H2170 cells (Fig. 2b, c). Furthermore, RIP assay also suggested that NNT-AS1 pull-down by Ago2 was significantly enriched in miR-22-transfected H1703 and H2170 cells (Fig. 2d, e). We also found that overexpression of NNT-AS1 inhibited miR-22 expression, while NNT-AS1 deletion promoted miR-22 expression in H1703 and H2170 cells (Fig. 2f, g). These data indicated that NNT-AS1 was a sponge of miR-22 and negatively regulated miR-22 expression in LUSC cells.

### NNT-AS1 deletion suppresses cell migration and invasion but induces cell apoptosis in LUSC by sponging miR-22

**Fig. 3:**
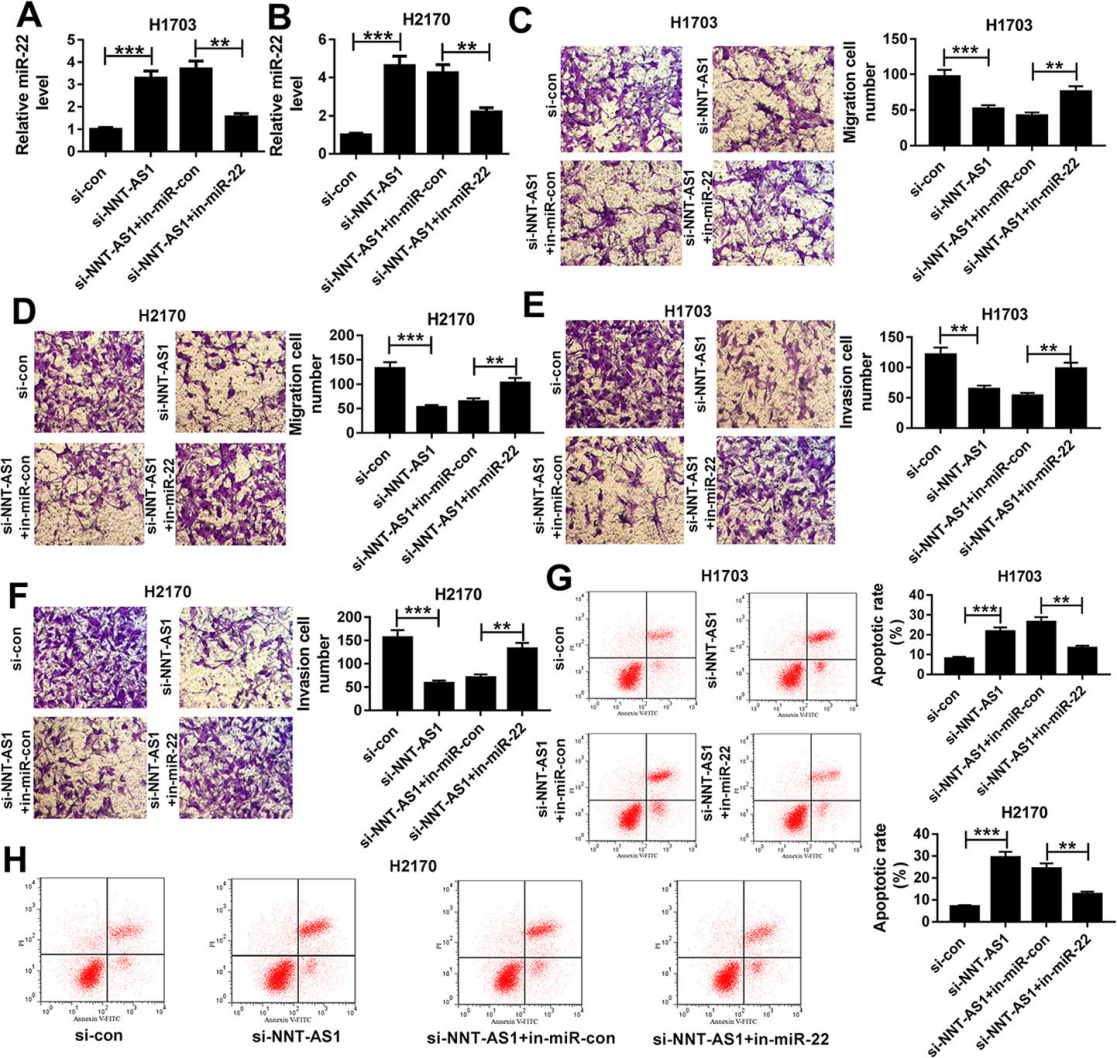
NNT-AS1 deletion suppresses the migration and invasion but induces apoptosis in LUSC cells by sponging miR-22. H1703 and H2170cells were transfected with si-con, si-NNT-AS1, si-NNT-AS1 + in-miR-con, or si-NNT-AS1 + in-miR-22. **a**, **b** Expression of miR-22 was measured using qRT-PCR. **c**-**f** Cell migration and invasion were determined by transwell assay. **g**, **h** Cell apoptosis was analyzed by flow cytometry. *P < 0.05, **P < 0.01, ***P < 0.001

Based on the relationship between NNT-AS1 and miR-22, we further wanted to explore the roles of the NNT-AS1/miR-22 axis in LUSC cells. Then H1703 and H2170 cells were transfected with si-con, si-NNT-AS1, si-NNT-AS1 + in-miR-con, or si-NNT-AS1 + in-miR-22. Meanwhile, the knockdown efficiency of in-miR-22 was detected and presented in Sup Fig. 1. Then, qRT-PCR results revealed that miR-22 expression was significantly elevated when NNT-AS1 was silenced, but this increase was attenuated after miR-22 inhibition in H1703 and H2170 cells (Fig. 3a, b). After that, transwell assay indicated that NNT-AS1 deletion inhibited the migration and invasion of H1703 and H2170 cells, while this inhibition could be reversed by the inhibition of miR-22 (Fig. 3c-f). Meanwhile, we also found that apoptotic cells were significantly increased by NNT-AS1 silence but were decreased by following miR-22 inhibition in H1703 and H2170 cells (Fig. 3g, h). Taken together, the evidence showed that NNT-AS1 deletion suppressed cell migration and invasion but induced cell apoptosis in LUSC by interacting with miR-22.

### FOXM1 is a target of miR-22

**Fig. 4:**
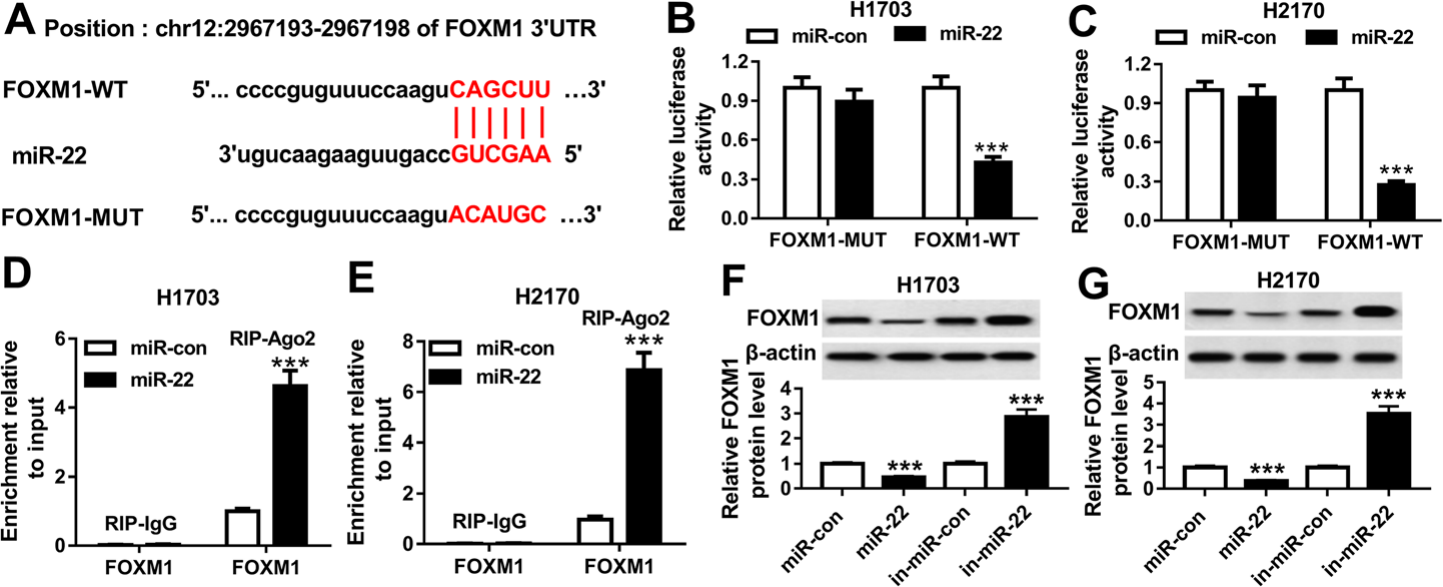
FOXM1 is a target of miR-22. **a** The binding site between miR-22 and FOXM1 is presented. **b**, **c** Luciferase activity was examined in H1703 and H2170 cells co-transfected with FOXM1-WT, or FOXM1-MUT and miR-22, or miR-con. **d**, **e** The interaction between miR-22 and FOXM1 was analyzed using RIP assay. **f**, **g** The level of FOXM1 was examined in H1703 and H2170 cells transfected with miR-con, miR-22, in-miR-con, in-miR-22 using western blot. *P < 0.05, **P < 0.01, ***P < 0.001

We further explored the molecular mechanisms by which the NNT-AS1/miR-22 axis regulated LUSC cell carcinogenesis, the potential target genes of miR-22 were screened using microT-CDS program and FOXM1 was identified to contain the putative binding site of miR-22 (Fig. 4a). Subsequently, a dual-luciferase reporter assay analysis showed that overexpression of miR-22 reduced the luciferase activities of the FOXM1-WT reporter vector, but not the FOXM1-MUT reporter vector inH1703 and H2170 cells (Fig. 4b, c). Moreover, RIP assay also proved the interaction between miR-22 and FOXM1 because of the obvious enrichment of FOXM1 in H1703 and H2170 cells (Fig. 4d, e). Additionally, we observed that the expression of FOXM1 was inhibited by overexpression of miR-22 but was promoted by miR-22 inhibition (Fig. 4f, g). Thus, these results showed that miR-22 interacted with FOXM1 to suppress its expression in LUSC cells.

### Overexpression of miR-22 inhibits cell mobility in LUSC by targeting FOXM1

**Fig. 5:**
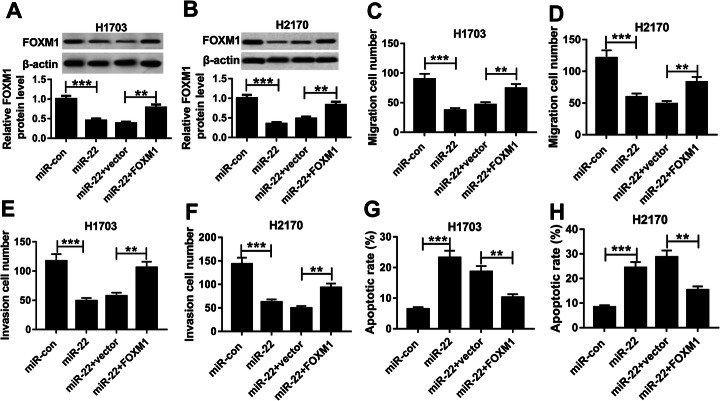
Overexpression of miR-22 inhibits cell carcinogenesis in LUSC by targeting FOXM1. H1703 and H2170 cells were transfected with miR-con, miR-22, miR-22 + vector, or miR-22 + FOXM1. **a**, **b** Western blot was used to detect the level of FOXM1 in H1703 and H2170 cells. **c**-**f** Migrated and invaded cells were detected by transwell assay. **g**, **h** Apoptotic cells were measured using flow cytometry. *P < 0.05, **P < 0.01, ***P < 0.001

According to the interaction between miR-22 and FOXM1, we further investigated whether the miR-22/FOXM1 axis was responsible for LUSC cell mobility. Synchronously, transfection efficiency of FOXM1 overexpression was detected and exhibited in Sup Fig. 1. Then H1703 and H2170 cells were transfected with miR-con, miR-22, miR-22 + vector, or miR-22 + FOXM1. After transfection, we found that the level of FOXM1 was inhibited by overexpression of miR-22, but was rescued by following FOXM1 restoration in H1703 and H2170 cells (Fig. 5a, b). Immediately, rescue assay was performed and the results indicated that overexpression of FOXM1 could reverse miR-22 restoration induced inhibition on cell migration and invasion (Fig. 5c-f), as well as promotion on cell apoptosis in LUSC (Fig. 5g, h). In all, we demonstrated that overexpressed miR-22 inhibited cell mobility in LUSC by directly interacting with FOXM1.

### NNT-AS1 indirectly regulates FOXM1 expression by sponging miR-22 in LUSC cells

**Fig. 6:**
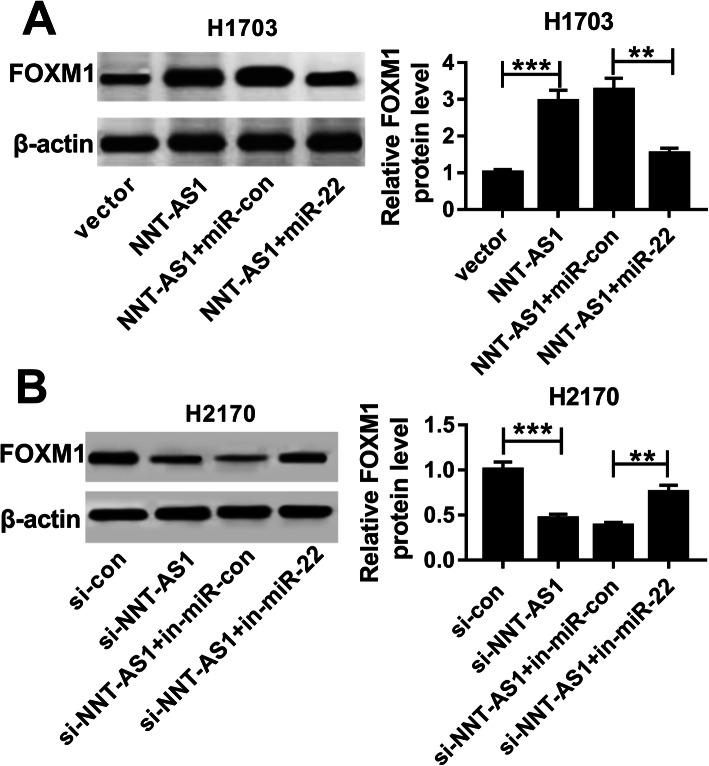
NNT-AS1 indirectly regulates FOXM1 expression by sponging miR-22 in LUSC cells. **a** Expression of FOXM1 was examined using western blot in H1703 cells transfected with vector, NNT-AS1, NNT-AS1 + miR-con, or NNT-AS1 + miR-22. **b** Expression of FOXM1 was determined by western blot in H2170 cells transfected with si-con, si-NNT-AS1, si-NNT-AS1 + in-miR-con, or si-NNT-AS1 + in-miR-22. *P < 0.05, **P < 0.01, ***P < 0.001

Based on the miR-22/FOXM1 axis, we further explored whether NNT-AS1 could regulate the FOXM1 expression by binding to miR-22. Subsequently, western blot analysis suggested that the level of FOXM1 was promoted by overexpression of NNT-AS1, while it was inhibited by following miR-22 restoration in H1703 cells (Fig. 6a). Meanwhile, we found that inhibition of miR-22 could rescue NNT-AS1 deletion induced inhibition of the expression of FOXM1 in H2170 cells (Fig. 6b). Altogether, these data suggested that NNT-AS1 functioned as a molecular sponge of miR-22 to regulate FOXM1 expression.

## Discussion

LUSC is the second most common type and accounts for roughly 30% of NSCLC. To date, molecular-targeted therapies have remarkably improved the overall survival of lung adenocarcinoma, but have little effect on the treatment of LUSC [23, 24]. Thus, there is an unmet need to develop novel therapeutics based on current genomic approaches for LUSC. LncRNAs are the principal elements of the mammalian transcriptome, which are emerging as master regulators in a wide variety of cellular processes, such as the cell cycle, differentiation, growth, and apoptosis, mediating the pathogenesis of many malignancies, including LUSC [25]. For examples, lncRNA HULC contributed to LUSC development by regulating the PTPRO/NF-κB signaling pathway [26]. LncRNA SNHG1 was over-expressed in LUSC tissues and promoted cell tumorigenesis in LUSC through regulation of ZEB1 by TAp63 [27]. Thus, lncRNAs may be useful therapeutic targeting in LUSC due to their multiple functions.

NNT-AS1, a novel cancer-related lncRNA, has been identified to function as an oncogene to participate in tumor cell development in many cancers [28, 29]. Recent studies have also revealed that NNT-AS1 was elevated in NSCLC, and dysregulated NNT-AS1expression was involved in the regulation of cell proliferation, invasion and cisplatin resistance in NSCLC [30, 31]. Here, we found that NNT-AS1 might also be involved in the development of LUSC. In this study, StarBase databases and results proved that NNT-AS1 was increased in LUSC tissues, indicating the potential regulatory roles of NNT-AS1 in LUSC. Functionally, NNT-AS1 deletion was identified to suppress cell migration and invasion, but induce cell apoptosis in LUSC. Taken together, the evidence indicated that NNT-AS1 functioned as an oncogene to promote cell tumorigenesis in LUSC.

In our study of the StarBase databases, we also found that miR-22 was up-regulated and FOXM1 was down-regulated in LUSC tissues compared to normal tissues. Moreover, the same changes of their expression were also observed in LUSC tissues and cell lines. Therefore, miR-22 and FOXM1 might be associated with the progression of LUSC. MiR-22 has been suggested to be frequently decreased, and exert an anti-tumor function in several cancers, including lung cancer [32]. Furthermore, miR-22 is a useful predictive biomarker and therapeutic target for lung cancer [33, 34]. In this study, miR-22 was also down-regulated in LUSC, and functional experiments showed that miR-22 overexpression inhibited cell migration and invasion, but induced cell apoptosis in LUSC. Moreover, our results confirmed that miR-22 was a target of NNT-AS1, and was negatively regulated by NNT-AS1 in LUSC tissues. The rescue assay also indicated that NNT-AS1 regulated cell carcinogenesis by binding to miR-22 in LUSC. Thus, an NNT-AS1/miR-22 regulatory axis was identified in the regulation of LUSC development.

FOXM1 belongs to the Forkhead box (Fox) family of transcription factors. FOXM1 is often expressed in actively dividing cells and plays important roles in cell cycle progression [35]. FOXM1 has been identified to be upregulated in lung cancer and is critical for the tumorigenicity of malignant lung cells, making it a new target for cancer diagnosis and therapies [36, 37]. Furthermore, FOXM1, as the downstream target, is frequently involved in the regulation of cancer progression [38]. This study verified that FOXM1 was a target of miR-22. Thus the miR-22/FOXM1 axis was investigated in LUSC. Subsequently, the rescue assay showed that overexpression of miR-22 inhibited cell mobility in LUSC by targeting FOXM1. Moreover, co-expression analysis suggested that NNT-AS1 could regulate FOXM1 expression by binding to miR-22 in LUSC cells. Therefore, an NNT-AS1/miR-22/FOXM1 regulatory network was identified in the LUSC.

In conclusion, our results demonstrated that FOXM1 was up-regulated in LUSC and overexpression of FOXM1 contributed to the tumorigenicity of LUSC cells. Additionally, molecular analysis showed that an NNT-AS1/miR-22/FOXM1 regulatory network in LUSC and NNT-AS1 silence exerted anti-tumor effects through the miR-22/FOXM1 axis in LUSC, providing a novel insight into the pathogenesis of LUSC.

## Supplementary information

**Additional file 1 **Sup **Fig. 1** Transfection efficiency of NNT-AS1 overexpression, si-NNT-AS1, miR-22 overexpression, in-miR-22, and FOXM1 overexpression was detected in LUSC cells.***P < 0.001.

## Data Availability

The data from this study are available in this published article.

## Abbreviations

LUSC: Lung squamous cell carcinoma
FOXM1: Forkhead box protein M1
NSCLC: Non-small cell lung cancer
FITC: Fluorescein isothiocyanate
PI: Propidium iodide
BCA: Bicinchoninic acid
ANOVA: analysis of variance
