# Supplementary figures and images for: LncRNA NNT-AS1 promotes lung squamous cell carcinoma progression by regulating the miR-22/FOXM1 axis
Source: Cell Mol Biol Lett. 2020 May 29;25:34. doi: 10.1186/s11658-020-00227-8 (PMC7257167; doi:10.1186/s11658-020-00227-8)

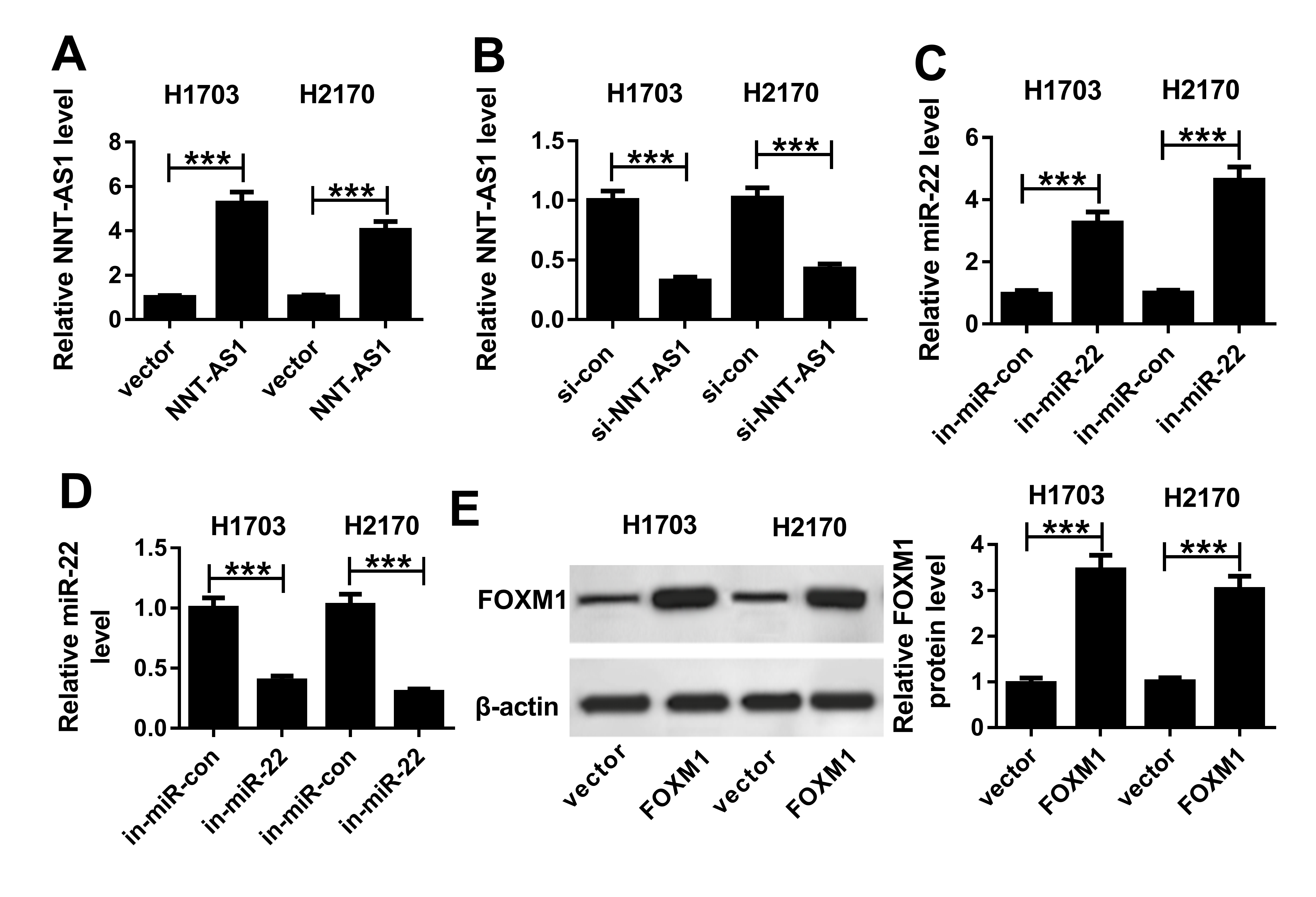

Supplement: Supplementary file 1 — Additional file 1 Sup Fig. 1 Transfection efficiency of NNT-AS1 overexpression, si-NNT-AS1, miR-22 overexpression, in-miR-22, and FOXM1 overexpression was detected in LUSC cells.***P < 0.001. [file 11658_2020_227_MOESM1_ESM.tif]
